# Lines of Action for Sexting Prevention and Intervention: A Systematic Review

**DOI:** 10.1007/s10508-021-02089-3

**Published:** 2021-11-17

**Authors:** Mónica Ojeda, Rosario Del Rey

**Affiliations:** 1 Department of Educational and Developmental Psychology, Faculty of Education Sciences, Universidad de Sevilla, C/ Camilo José Cela, s/n, 41018 Sevilla, Spain; 2 Department of Educational and Developmental Psychology, Faculty of Education Sciences, Universidad de Sevilla, C/ Pirotecnia, 19, 41013 Sevilla, Spain

**Keywords:** Sexting, Childhood, Adolescence, Lines of action, Systematic review

## Abstract

Sexting has become a new form of intimate interaction in line with contemporary communication methods. This phenomenon often leads to positive outcomes, but it can also have negative repercussions depending on the situation, such as the context of the relationship, and whether it is consensual or coercive. Despite this, the main types of sexting behaviors (sending, receiving, and third-party forwarding) must be addressed in order to promote safe and healthy practices. However, the approach to tackling this phenomenon remains unclear. This systematic review sought to summarize the lines of action proposed or conducted in the scientific literature to address sexting, to help researchers and educators create and evaluate effective programs. A systematic search of 21 databases was conducted; only articles relating to sexting education, prevention, and intervention among child and adolescent populations were considered. In total, 456 articles were identified, 91 of which were included for the purposes of this research. The results highlighted a need to respond to the aforementioned sexting behaviors and to tackle the resulting conflict situations. Although interventions across different areas are recommended (e.g., health, family, policies, legal advice, law enforcement, technology experts, and even society as a whole), most studies agree that school is the most practical setting for intervention. Thus, the 15 lines of action identified in this systematic review must all be considered to effectively address sexting in childhood and adolescence.

## Introduction

Social networks and digital media have become a major part of our daily lives, exerting an increasingly significant impact on individuals in general and, more specifically, on adolescents (Smith et al., 2016). Digitally driven communication fosters positive interactions and has multiple benefits, but it can also bring about new challenges (Englander & McCoy, 2017). The emergence of online communication has been linked to the global rise in messages with sexual content (Sweeny & Slack, 2017). Today, adolescents can explore their sexuality in new ways, redefining and normalizing more recent types of intimate relationships such as sexting—the sharing of self-produced sexual material through electronic means (Barrense-Dias et al., 2017; Schubert, 2014).

Concerns about this phenomenon have grown, and it has attracted considerable attention from researchers, families, teachers, schools, and the media (Anastassiou, 2017; Gewirtz-Meydan et al., 2018; Van Ouytsel et al., 2015). This has occurred as a consequence of its potential negative impact and the effects it can have on adolescent well-being, where sexual content is disseminated without consent or where teens feel peer or partner pressure to engage in sexting behaviors (Klettke et al., 2014; Olivari & Confalonieri, 2017; Schubert, 2014). Even teachers agree that sexting could cause classroom disruption (O’Bannon & Thomas, 2014).

The sexting phenomenon may play an influential role in the process of building new relationships as well as in the development of adolescents’ sexual behaviors (Ringrose et al., 2012). Therefore, identifying the institutions that need to get involved and the types of actions that need to be taken is key to ensuring effective prevention and intervention in these areas. Thus, this systematic review analyzes the scientific evidence that helps identify the lines of action to address sexting.

### Definition and Prevalence of Sexting

There is a lack of general consensus around the definition of sexting. Definitions vary depending on the type of behavior included, alluding to active sexting (such as sending or third-party forwarding) and passive sexting (receiving a message directly from the creator or via an intermediary). They also vary according to the content of the messages (sexual pictures, videos, or text) and the degree of sexual explicitness (suggestive or explicit) (Barrense-Dias et al., 2017). Therefore, the most restrictive definitions exclusively refer to sending sexually explicit pictures (Choi et al., 2016; Marume et al., 2018), whereas the most comprehensive definitions also cover other types of behavior, for example, content dissemination (Mitchell et al., 2012; Villacampa, 2017). In this context, sexting encompasses the sending, receiving, and forwarding of suggestive and explicit sexual pictures, videos, or text messages via cell phones, the Internet, or other electronic means (Mitchell et al., 2012). Because sexting is an evolving concept that has become increasingly complicated (Van Ouytsel et al., 2018), each study and the sexting behavior must be analyzed, as the action to be taken may vary depending on the behavior displayed.

The estimated number of adolescents engaging in sexting is consistently definition dependent (Barrense-Dias et al., 2017). In a recent meta-analysis (Madigan et al., 2018), the average prevalence of sending sexual content was 14.8%; receiving sexts was 27.4%; forwarding a sext without consent was 12.0%; and receiving a forwarded sext was 8.4%. In recent years, sexting rates among youth have seen a rise with increasing age, and no significant gender differences in the rate of sending or receiving sexts have been observed (Madigan et al., 2018).

### The Need to Address Sexting

The practice of sexting is characterized by its psychological, social, and behavioral consequences (Klettke et al., 2014), which can lead to ethical and socio-moral conflicts alongside other concerns about the privacy and protection of personal content (Schubert, 2014).

Results of a recent meta-analysis suggest that the exchange of sexual messages, photographs, and videos through technological devices is associated with sexual behavior (sexual activity, multiple sexual partners, lack of contraception use, etc.) and mental health issues (delinquent behavior, anxiety/depression, alcoholism, drug consumption, smoking, etc.), especially in younger adolescents (Mori et al., 2019). The consequences of sexting may affect the physical and psychological health of those involved, and adolescents may end up experiencing peer pressure and emotional difficulties (Olivari & Confalonieri, 2017; Van Ouytsel et al., 2015). However, most studies indicate that these relationships are cross-sectional, and the fact that sexting presents as a problematic behavior would seem to depend on the situation, such as the context of the relationship, and whether it is consensual or coercive (Temple et al., 2019).

Although the bulk of the research focuses solely on consented parties (sending and/or receiving this type of content), the most detrimental action, and therefore the most important when trying to understand the consequences behind this phenomenon, would be the forwarding of sexual content by third parties (Livingstone & Görzig, 2014; Strassberg et al., 2017). A possible explanation is that sexual content can be spread quickly without consent, reaching undesired recipients, thus increasing its audience and affecting the victim’s reputation (Van Ouytsel et al., 2014a, 2014b). Consequently, most efforts should be directed in this area to prevent and effectively intervene in sexting behaviors. As a result of this dissemination, sexting is also associated with other potential risks which can aggravate its possible consequences, such as blackmail, extortion, bullying, and cyberbullying (Döring, 2014; Kopecký, 2015; Medrano et al., 2018; Montiel et al., 2016; Strassberg et al., 2013; West et al., 2014; Woodward et al., 2017).

The gender dynamics that arise from this phenomenon are also noteworthy. Differences in the roles of sexting are observed, and the different practices do not seem to affect boys and girls in the same way. Boys are perceived as those who ask for photographs, whereas girls are seen as those responsible for setting the boundaries (Symons et al., 2018). Moreover, girls usually experience a damaged reputation and tend to suffer the consequences of sexting more than their male peers, the latter even experiencing positive effects which can boost their popularity (Cooper et al., 2016; Dobson & Ringrose, 2016; Symons et al., 2018; Wood et al., 2015).

In all cases, a robust response to any sexting-related behavior (sending, receiving, and third-party forwarding) is highly recommended. It is clear that the non-consensual forwarding of content to third parties is a type of behavior that must be avoided, and prevention strategies must be taught (Van Ouytsel, et al., 2014a, 2014b). However, young people also need to know how to act when this type of content is received (Mitchell et al., 2012); how to send sexual content safely, if this is indeed the intention; and how to fend off any attempts at peer pressure (Wurtele & Miller-Perrin, 2014).

Thus, this phenomenon is seen as a challenge for educational institutions and teaching professionals (McEachern et al., 2012), given that incidents brought about by sexting can have a negative impact at school (Van Ouytsel et al., 2014a, 2014b, 2015). Although prevalence is higher outside of school walls, sexting also occurs during school hours (Schubert, 2014). What is more, sexting outside of this environment may also have an impact within the school context (McEachern et al., 2012). Because this phenomenon has the power to influence the interpersonal relationship building process, not to mention adolescent sexual development in general (Ringrose et al., 2012), the classroom is an ideal environment for educating individuals on how best to use Information and Communications Technology (ICT) and, more specifically, prevent any negative consequences of sexting (Mura et al., 2014; Theodore, 2011). Educating young people about sexting in schools has the potential to reach far more adolescents, as many young people do not learn about sexuality or this new reality at home. Furthermore, teaching professionals can train and inform parents, fostering positive relationships and building a strong school community that guarantees the continuity of education received by students in both key settings: home and school (Van Ouytsel et al., 2014b). Specifically, teaching professionals can play a crucial role when it comes to addressing this phenomenon proactively and when taking specific preventive actions to address the consequences of sexting (Bhat, 2018; Kopecký, 2012).

### Research Question

Despite the importance of preventing the potential negative consequences of sexting (Van Ouytsel, et al., 2014a, 2014b), information on how to do this effectively remains scarce. As such, there is an obvious need to develop strategies based on scientific research findings (Livingstone & Smith, 2014), identifying areas and lines of action that can help researchers and educators create and evaluate programs to successfully address sexting. In an effort to bridge this gap, this study aims to systematically describe the available scientific evidence outlining the effective lines of action to tackle sexting. To this end, the present review is guided by the following question: What are the types and frequencies of proposed lines of action for sexting?

## Method

### Inclusion and Exclusion Criteria

This systematic review includes all articles published up until 2018 that meet a predetermined set of inclusion and exclusion criteria.

The specific criteria to determine suitable studies for inclusion in the review were:

a Studies with a target or participant population of children or adolescents aged up to 19 years.
b. Studies that consider sexting as their phenomenon of interest.
c. Studies that include information about sexting education, prevention, and/or intervention.

The specific criteria to determine studies that should be excluded from the review were:

- Studies with an adult target or participant population.
- Studies whereby the topic of interest is a sexual phenomenon other than sexting, such as grooming, sexual abuse, or pornography.
- Duplicate articles.
- Articles with insufficient information because the full text is not available.

### Search Strategy

The search strategy used for this systematic literature review was based on the PRISMA statement (Urrútia & Bonfill, 2010). The studies were collected from the following 21 databases: Scopus, Web of Science, Dialnet, CSIC, Periodicals Archive Online, SportDiscus, Psicodoc, ERIC, PsycINFO, Sociological Abstracts, PsycArticles, PubMed, Social Service Abstracts, PILOTS, Redalyc, PubPsych, Teacher Reference Center, Science Direct, Elsevier, ACM Digital Library, and IEEE Xplore.

The key words used were *sexting*, *child*, *minor*, *adolescent*, *teen*, *youth*, *student*, *prevention*, *education*, and *intervention*. The following search query was entered for the title, abstract, and key words: “Sexting AND (child* OR minor OR adolesc* OR teen* OR youth* OR student*) AND (prevention OR education OR intervention).” Before selecting this strategy, other attempts were made, such as “sexting AND adolesc* AND (prevention OR intervention).” The results were examined to find the right balance between sensitivity and specificity. Database searches were conducted up to September 2018.

### Data Coding and Analysis

Article coding was carried out in two phases using an analysis sheet. During the first phase—the abstract screening phase—a check was run to ensure that the article abstracts met the inclusion criteria and not the exclusion criteria. To do so, the following data were collected: database, year of publication, authorship, journal/publication, article title, inclusion criteria (a) and (b), and exclusion criteria (a), (b), and (c).

The second phase—full-text eligibility—was carried out on articles that only met the previous inclusion criteria. Checks were run to verify that they also met inclusion criterion (c) and that they did not meet exclusion criterion (d). In this case, the whole publication was analyzed: type of study, area of study (journal/editorial field of study), language, country (country of the participating sample or, if not, the first author's home institution), objective(s), methodology, recipients, sample, definition of sexting, instrument, instrument characteristics, evidence of action, type of action, evaluation of action, area of action, and results. Only those articles clearly stating their own definition of sexting were taken into account. Review articles describing the definitions adopted in other articles without taking a personal stance were not considered. In the case of review articles, only recommendations pertaining to the article were considered, and proposals collected from the analyzed articles were discarded.

Coding was undertaken by the principal investigator. Subsequently, 57.2% of the articles were selected at random and codified equally and independently by a second assessor, easily exceeding the 20% recommended minimum (García-Moya et al., 2018). During this phase, the sexting definitions found in the articles under study were also categorized, leading to the following category system: specific behaviors by definition (distribution/exchange; sending; sending and receiving; sending, receiving, and forwarding), the type of content (text message; pictures; text messages or pictures; pictures or videos; text messages, pictures or videos; not specified), and the degree of sexual explicitness (explicit; suggestive or explicit; not specified).

The reliability level was high. The percentage of agreement in the abstract screening phase was 91.3%, reaching 92.1% in the full-text eligibility phase. Disagreements were discussed and resolved in a consensual manner. The articles selected were downloaded using the Mendeley 1.19.3 software program.

**Fig. 1:**
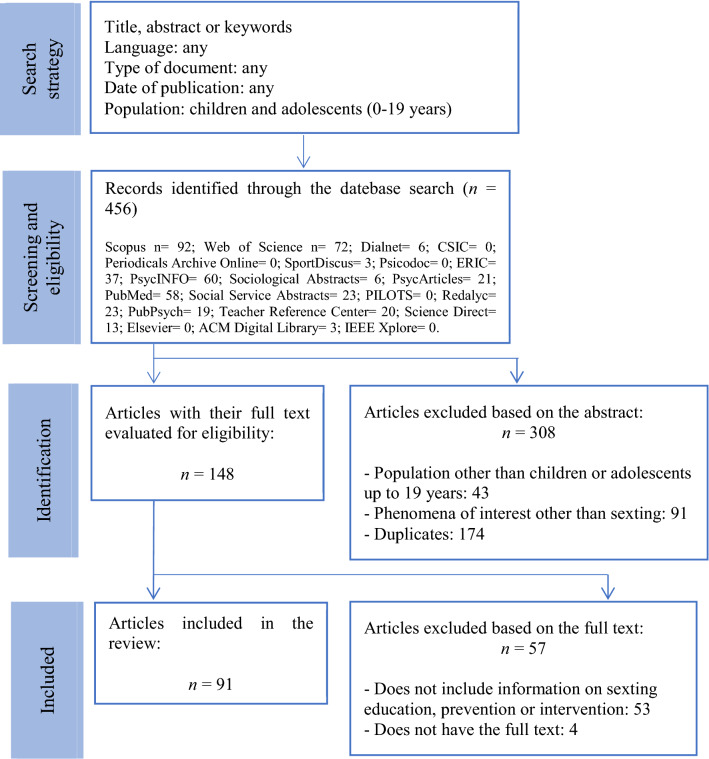
Review of the article selection process

Figure 1 shows a summary of the selection process. A total of 456 articles were identified for the systematic review. The abstract was taken into account across all reviews, and 308 articles were excluded as they met exclusion criteria (a), (b), or (c). The full text of the remaining articles was analyzed, and 57 articles were excluded as they did not meet inclusion criteria (c) or they met exclusion criteria (d). Therefore, 91 articles were included in this systematic review. An overview of the general characteristics corresponding to these studies is provided in Appendix.

Once the included articles had been determined, the areas and lines of action found in each article were categorized. The category system resulting from categorizing the areas of action is shown in Table 1. Similarly, the category system resulting from categorizing the lines of action is shown in Table 2.

**Table 1:** Areas of action

	Description	N	%
1. School	School counselors, educational psychologists, school nurses, teachers, and centers of education in general	79	86.8
2. Family	Fathers, mothers, guardians	19	20.9
3. Family through school	The family’s involvement through the school. This refers to situations in which families are encouraged to engage in school-led actions. The goal is that families take action, too	19	20.9
4. Health	Pediatricians, doctors, nurses, gynecologists, and psychologists	18	19.8
5. Policies	Politicians, legislators	12	13.2
6. All areas	In general, or referring to the community or society as a whole	7	7.7
7. Family through health	The family’s involvement through the health sector. This refers to cases in which health professionals engage the family. The goal is that families take action, too	5	5.5
8. Legal advice	Specialists in the legal sector (legal advisers, legal point of view)	5	5.5
9. Law enforcement	Law enforcement authorities	4	4.4
10. Technology experts	Web designers	2	2.2

**Table 2:** Lines of action identified to address sexting

	Description	N	%
1. Developing specific sexting programs	The implementation of training/awareness activities and programs that address sexting in a specific way (definition, characteristics, reasons for participation, coping strategies, possible consequences, how to carry out safe sexting, etc.). These can be undertaken as face-to-face activities and/or through ICT	41	48.8
2. Promoting safe and healthy use of ICT, the Internet, and social networks	The development of activities and programs to provide strategies that encourage safe online behaviors	39	46.4
3. Raising awareness about the consequences and risks of sexting	The need to discuss and analyze the specific consequences and risks that sexting can bring	29	34.5
4. Incorporating information about sexting into sex education programs	The integration of sexting as another form of sexual behavior through digital media into training/awareness activities and sex education programs. These can be undertaken as face-to-face activities and/or through ICT	26	31.0
5. Training professionals	Continuous training and ongoing development for professionals who work with minors and young people	24	28.6
6. Promoting sexual ethics	Fostering the necessary skills to make ethical decisions regarding intimate relationships	20	23.8
7. Raising awareness about gender roles and stereotypes	Analysis of gender roles and stereotypes, challenging the heteronormative rules of femininity and masculinity	17	20.2
8. Developing rules and implementing protocols	Drawing up clear school rules that regulate possible conflictive situations related to new technologies and sexting, and designing protocols to help professionals know how to react and to tackle conflictive situations concerning sexting	16	19.0
9. Encouraging coherence between the different parties involved	The need to involve the education community as a whole in the actions taken to address sexting, as well as other institutions and society in general, whenever possible	15	17.9
10. Working on the risk factors associated with the peer group	Taking actions that address the importance of peer culture and the role of spectators	13	15.5
11. Considering the ideas and experiences of adolescents	The need to build on the perceptions and practices held firsthand by adolescents, and to integrate them into the sexting-related actions to be taken. This can be carried out all together or in groups divided by gender	13	15.5
12. Improving the school environment	Encouraging positive, caring, and respectful relationships among the education community	10	11.9
13. Developing measures adapted to vulnerable groups	The design and implementation of specific activities and strategies aimed at different groups who have shown a higher probability of being harmed, such as the LGBTQIA + community, ethnic minorities, students with a high risk of online victimization or greater impulsiveness, etc.	9	10.7
14. Applying disciplinary or legal measures, if needed	Establishing clear school behavioral sanctions that the education center deems negative and identifying criminal offense situations as cases where sexual content is created/distributed without consent	8	9.5
15. Incorporating sexting into preventive programs that tackle other associated risks	The incorporation of training strategies on sexting in the activities and programs that address the different risks associated with this phenomenon	7	8.3

The sum of the areas, lines of action, and relevant information found in the articles were also categorized by an external reviewer. The reliability level was high (percentage of agreement at 84.2%). Disagreements were discussed and resolved in a consensual manner.

## Results

### Overview of the General Characteristics of the Studies

The general characteristics analyzed in the articles were: year of publication, geographical area of origin, subject area, definition of sexting, type of action recommended, and area of action where intervention is deemed necessary.

Regarding year of publication, the reviewed articles were published between 2009 and 2018, as no articles pre-2009 were found on any of the 21 databases. Notably, an increase in publications was observed in 2014; however, wide frequency variability is found and a clear pattern cannot be determined.

In terms of geographical area of origin, 52.2% of studies were conducted in North America, 27.8% in Europe, 10% in Oceania, 4.4% across several continents, 3.3% in South America, 1.1% in Africa, and 1.1% in Asia.

Regarding subject area, 39.6% of studies were conducted in the field of health, 26.4% in psychology, 20.9% in other social science disciplines, 11% in education, 8.8% in sociology and political sciences, 4.4% in the field of communication, and 3.3% in the discipline of law.

The definition of sexting varies depending on the specific behavior at play, the type of content, and the degree of sexual explicitness. In terms of the different behaviors, 39.2% of articles only refer to sending, 23% generally define sexting as a sharing or exchange process, 17.6% refer to sending and receiving, and 16.2% identify the three behavior types: sending, receiving, and forwarding. The type of content behind these messages also varies. Specifically, 29.7% of articles mention text messages, pictures, and videos; 29.7% refer only to pictures; 28.4% refer to text or picture messages; 6.8% refer to sexual content in general but do not specify the content; 2.2% refer to pictures or videos; and 2.2% refer only to text messages. Lastly, 48.7% include suggestive and explicit content; 43.2% include only explicit content; and 8.1% do not specify the type of sexual content behind the definition used (Table 3).

**Table 3:** Definition of sexting

	N	%
Behavior		
Sending	29	39.2
Sharing/exchanging	17	23.0
Sending and receiving	16	21.6
Sending, receiving, and forwarding	12	16.2
Type of content		
Pictures	22	29.7
Text messages, pictures, or videos	22	29.7
Text messages or videos	21	28.4
Does not specify	5	6.8
Pictures or videos	2	2.7
Text messages	2	2.7
Degree of sexual explicitness		
Suggestive or explicit	36	48.7
Explicit	32	43.2
Does not specify	6	8.1

In terms of the type of action recommended, 7.7% of articles recommend taking actions to address and prevent the potential consequences of sexting, but do not include any interventions or suggest any strategies to make this happen; 85.7% of articles do propose specific strategies that can be effective when addressing this phenomenon, but do not include any interventions; and 6.6% present specific interventions to tackle sexting.

Regarding the area of action where intervention is required, 86.8% of articles indicate that intervention should be school-led. Next, 20.9% state that the family should intervene, whereas the same percentage of articles (20.9%) report that family intervention must also involve the school. Similarly, 19.8% state that action should be taken from a healthcare perspective; 13.2% from a political perspective; and 7.7% from across all areas in general. A total of 5.5% of articles state that family-led involvement should be health based, whereas the same percentage (5.5%) report how legal advice is required to lend a legal perspective on this phenomenon. Moreover, 4.4% of articles call for the participation of law enforcement agencies, and 2.2% note that collaboration from technology experts is also necessary (Table 1).

### What are the Types and Frequencies of Proposed Lines of Action for Sexting?

The collected data have been divided into three different types of information: lines of action, initiatives developed, and main recommendations.

Fifteen lines of action emerged after categorizing the strategies identified in the research papers as key aspects of tackling sexting (Table 2):

A total of 48.8% of articles recommend developing specific sexting programs; 46.4% encourage a safe and healthy use of ICT, the Internet, and social media; 34.5% recommend raising awareness about the consequences and risks associated with sexting; and 31% set out the need to incorporate sexting into sex education programs. Furthermore, 28.6% recommend training professionals; 23.8% recommend promoting sexual ethics; 20.2% highlight the need to raise awareness about gender roles and stereotypes; and 19% advocate developing rules and implementing protocols. A total of 17.9% of articles encourage coherence among the different parties involved; 15.5% set forth the need to work on risk factors associated with peer groups; 15.5% call for the ideas and experiences of adolescents to be heard and used; and 11.9% recommend improving the school environment. Furthermore, 10.7% of articles suggest developing measures adapted to vulnerable groups, and 9.5% highlight the need to apply disciplinary or legal measures. Lastly, 8.3% recommend integrating sexting into preventive programs about other associated risks.

In addition to these lines of action, only six articles set out specific initiatives to address sexting: the specific action under the 3rd Spanish Master Plan for the Coexistence and Improvement of School Safety; the Webrangers educational project; the action research project Image.me; a three-level strategic plan; the Sextorsion prevention course; and school assemblies about the risks of sexting.

The specific action established under the **3rd Spanish Master Plan for the Coexistence and Improvement of School Safety** by the Spanish Government’s Ministry of Education, Culture, and Sport; the Ministry of the Interior; and the Ministry of Public Health and Social Affairs contains presentations about the safety issues and risks associated with the use of the Internet to be developed alongside high school students. A Spanish civil guard officer, who is a specialist in New Technology and Risks, delivered an hour-long presentation about the possible risks of Internet use, especially those derived from using social networking sites, such as cyberbullying, grooming, and sexting. After the presentation, students completed an ad hoc questionnaire about their social network involvement. Information was gathered about their usage time or external supervision; the presentation content they found most interesting; and their personal opinions about the role of the Spanish civil guard officer. The impact of the activity on sexting was not evaluated (Martín et al., 2013).

The **Webrangers** education project is delivered in partnership with Google Inc., Palacký University Olomouc, and the NGO Google Education Group. It consists of a peer training program in which students interested in the topic are selected and given full-time training. This project covers risky Internet behaviors and the safe use of this tool to help prevent dangerous online conduct. Case studies are used to train students on the following core topics: cyberbullying, cyber-grooming, sexting, and skills for online interaction. After the training, students must create their own projects to raise awareness through Google Plus, Facebook, the project’s Web site, and through workshops and activities for their colleagues and teachers. Information about their evaluation was not reported (Kopecký et al., 2015).

**Image.me** is an action research project about sexting prevention. It uses social theater with young people as preventive medical care, focusing on peer education through media and digital literacy. This combination encourages critical thinking and promotes collaborative work between classmates. These activities are part of a wider research project about theater and scientific communication led by the Catholic University of Milan. Social theater was used as a form of social care and online risk prevention. Three art scenes tailored to the project’s target audience were chosen in an attempt to envisage how to address sexting effectively. One of the results was the creation of a pet-puppet used to get to know young people at schools, clubs, and other informal contexts. Videos were also made to raise young people’s awareness about the presence of sexting in their communities. Social theater was also used to communicate the research findings at the end of the project. An art scene was designed and used to discuss the information gathered, making it easier to understand and engage with. Information on its evaluation was not reported (Ferrari et al., 2016).

The **three-level strategic plan** describes specific activities that schools can implement to tackle cyberbullying, sexting, and other risk behaviors on social media. The three-level model aims to meet the common needs of all students, the specific needs of some students, and other more specific and complex needs. Level 1 meets the general needs. It aims to provide a definition of the phenomenon and set out regulations planned for and by the education community, which also covers how to handle a conflictive sexting situation. In addition, as part of the school curriculum, the whole institution should be given training about this phenomenon and its effects in order to safely address any kind of online behavior. Assessment twice a year is also important. Level 2 offers strategies for students at risk of becoming a bully or a victim. Prevention groups are formed to work on specific skills according to the potential participants’ needs. Finally, Level 3 is delivered to students who are already actively involved in sexting and have more complex needs. In this case, intervention should focus on their direct needs, such as individual advice geared toward specific abilities, meetings with family members, and disciplinary and/or legal action. In general, the activities need to stress the importance of educating those involved about the Internet and its dangers. Information about its evaluation was not reported (Davis & Schmidt, 2016).

The **Sextorsion prevention course** is delivered in high schools to teach students how to practice safe sexting. The learning methodology combines traditional lectures which provide an overall description of the topic with active learning, and directly engages students in the learning process. The course content includes the conceptualization and characteristics of sexting and sextortion, its associated risks, empathy toward the victim, legal consequences, safe practices, and measures and protocols to deal with the phenomenon. In particular, active learning was carried out by simulating different sexting and sextortion scenarios, encouraging students to be more independent and building their ability to search for relevant information related to sexting and sextortion. Different resources such as videos, group discussions, cases analyses and simulations, and group reflections were used. Regarding assessment, students answered a pre- and post-course questionnaire about their knowledge of the topic and how satisfied they were with the course. However, the impact of the activity was not reported (Palop et al., 2016).

Finally, **school assemblies** were designed to educate high school pupils on the risks of sexting. The specific content of these assemblies was not provided. However, four years later, sexting patterns (except third-party forwarding) had not changed significantly. Most adolescents had exchanged sexually explicit pictures on their phones, and the common behavioral narratives remained very similar (Strassberg et al., 2017).

The previously stated lines of action and initiatives were joined by other notable recommendations for tackling this phenomenon:

Specifically, 19% of articles recommend staying away from scare tactics as a tool for intimidating young people; 11.9% recommend avoiding messages that promote the abstinence from and prohibition of sexting given their low level of effectiveness, adopting a more educational than authoritarian perspective; and 9.5% recommend not blaming and judging the victim or those who partake in this practice. Furthermore, 9.5% of articles highlight the need to start taking action early on in school and in preadolescence, and 7.1% recommend sexting assessment in schools to establish a baseline and to be able to promote strategies and actions based on the results obtained. Finally, 11.9% of articles recommend evaluating the impact of these strategies post-implementation to determine their effectiveness. Thus, the practices could be evidence based, promoting continuous improvement and adapting the strategies to the intervened context.

## Discussion

Sexting has shaped itself into a new form of adolescent sexual exploration and expression (Schubert, 2014). However, it has also become a new challenge that professionals working with children need to understand in order to deal with it effectively (Bhat, 2018; Kopecký, 2012). This systematic review sought to gather information and describe the existing scientific evidence relative to the effective lines of action that address sexting, helping researchers and educators to design and evaluate sexting programs.

There is scientific evidence to support the need for sexting intervention. Specifically, these efforts must focus on the different ways in which this phenomenon is experienced and expressed: sending, receiving, and forwarding. The most commonly used sexting definition in the analyzed articles had sending as the most studied behavior. However, this definition does not cover the phenomenon’s complexity. Third-party forwarding of sexual content also plays a highly significant role in understanding the consequences of sexting (Livingstone & Görzig, 2014; Strassberg et al., 2017). Thus, it is important to include all three types of sexting behaviors (sending, receiving, and third-party forwarding) in order to analyze each one on its own and to be able to identify the necessary actions for each behavior.

Studies mainly focus on a particular geographical area (North America) and a specific subject area (Health), meaning that sexting research in other countries and in the educational field must also be encouraged. At a disciplinary level, the focus is primarily placed on the school setting (Livingstone & Smith, 2014; Van Ouytsel et al., 2014b). Furthermore, only six of the 91 articles feature a specific intervention and just one article evaluates the impact of this action. This may be due to the fact that literature and educational campaigns have mainly focused on analyzing sexting as a problem, promoting abstinence, and condemning the practice. Consensual sexting as an intimate means of communication in line with contemporary communication methods must be accepted (Döring, 2014; Strassberg et al., 2017).

### What are the Types and Frequencies of Proposed Lines of Action for Sexting?

Fifteen lines of action to address sexting effectively have been identified: (1) developing specific sexting programs; (2) promoting safe and healthy use of ICT, the Internet, and social networks; (3) raising awareness about the consequences and risks of sexting; (4) incorporating information about sexting into sex education programs; (5) training professionals; (6) promoting sexual ethics; (7) raising awareness about gender roles and stereotypes; (8) developing behavioral rules and implementing protocols; (9) encouraging coherence between the different parties involved; (10) working on the risk factors associated with the peer group; (11) considering the ideas and experiences of adolescents; (12) improving the school environment; (13) developing measures adapted to vulnerable groups; (14) applying disciplinary or legal measures, if needed; and (15) incorporating sexting into preventive programs that tackle other associated risks.

The development of specific programs that address sexting is the notable line of action. Undertaking both proactive and reactive activities is crucial (Albury et al., 2017). Some examples are: the use of case studies (e.g., Kopecký, 2015; Palop et al., 2016); discussions (e.g., Gregg et al., 2018; Siegle, 2010); educational campaigns, lectures, and workshops (e.g., Dobson & Ringrose, 2016; Hinduja & Patchin, 2012); the creation of information resources, a compilation of best practices (e.g., Döring, 2014; McEachern et al., 2012); real testimonies (Martín et al., 2013; Van Ouytsel, et al., 2014a, 2014b); debates (e.g., Dobson & Ringrose, 2016; Van Ouytsel et al., 2015); and cross-curricular classroom projects (Laguado et al., 2018; Theodore, 2011).

Fostering a safe and healthy use of ICT, the Internet, and social networks is also noteworthy. Because adolescents who use their cell phones as their main Internet connection and spend more time connected are most likely to receive sexting requests (Atwood et al., 2017), it is important to teach them how to use technology appropriately. This training should cover personal expectations about digital privacy (e.g., Albury et al., 2017; Soriano-Ayala & González-Jiménez, 2014); control over personal data on the Internet (e.g., Diliberto & Mattey, 2009; Patrick et al., 2015); safe online behaviors (e.g., Mura et al., 2014; O’Keeffe, 2016); and knowledge of rights and responsibilities when it comes to digital technology (e.g., Gámez-Guadix et al., 2017; Uhler & Smith, 2012).

Efforts to incorporate sexting into sex education programs is also a fundamental part of handling this phenomenon, as sexting may be seen as a way to maintain intimate communication with a partner in a healthy relationship (Van Ouytsel, et al., 2014a, 2014b). On many occasions, sexting is used to show a romantic or sexual interest in another person; to build new emotional bonds; to delve deeper into the development of their sexual identity; and merely as another form of sexual activity in a long-distance relationship (Döring, 2014; Walker et al., 2011). Thus, addressing sexting as an integral component of sex education programs provides young people with information about the phenomenon and how to tackle it safely, instead of evading it or encouraging the negative views held by many adolescents about sexting. Given the correlation between sexting and traditional or digital risks, such as bullying and cyberbullying (e.g., Rodríguez-Castro et al., 2017; Woodward et al., 2017), introducing sexting into preventive programs that address other associated risks—adopting an integrated approach—is also recommended (e.g., Dake et al., 2012; West et al., 2014).

The promotion of sexual ethics is also linked to sex education, namely specific and key aspects that address sexting effectively. Its focus is on developing the necessary skills to build and maintain an intimate and ethical relationship (Walker et al., 2011), such as preventing coercion and pressure in a loving sexual relationship; fostering reflection on the importance of proper consent and real respect for a partner or intimate companion; and maintaining a critical attitude toward the exchange of non-consensual sexual content (e.g., Albury et al., 2017; Wurtele & Miller-Perrin, 2014). In terms of sexual ethics, gender roles and stereotypes must also be considered. Acknowledging the cultural norms and values that underpin social behavior is essential to successfully addressing the phenomena which play out in personal interactions. Thus, it is necessary to understand and question the heteronormative values associated with femininity and masculinity which form part of the digital culture and to determine the dynamics and roles played by individuals who engage in sexting (e.g., Karaian, 2014; Wood et al., 2015). It is particularly important to involve young people in analyzing the power imbalance between genders and the double sexual standard and to avoid the use of stereotypes and blaming women, in the strategies used to address sexting (e.g., Döring, 2014; Van Ouytsel et al., 2014a).

Raising awareness about the impact and risks of sexting is also important. Sexting can lead to undesired consequences, which can turn into problematic scenarios alongside other risks such as bullying and cyberbullying (Frankel et al., 2018; Medrano et al., 2018; Ringrose et al., 2012). For this reason, young people need to be aware of the risks. However, we need to remember that, for some people, sexting is a romantic and enriching part of their relationship, although it can be dangerous (Ybarra & Mitchell, 2014). Furthermore, some studies suggest that many adolescents already have a clear picture of the consequences this phenomenon entails, and steps to ban and warn against sexting alone do not work to prevent the potential consequences (Lim et al., 2016). In this systematic review, only one evaluation of the proposed lines of action has been observed, which focuses on the lack of efficacy behind this strategy. It does, however, seem to reduce the extent to which this type of content is forwarded without consent, yet it does not effectively avert other possible negative consequences linked to sexting (Strassberg et al., 2017).

Providing training to professionals who work with young people would also help them feel more capable of addressing sexting. It would equip them with the skills to react properly to difficult situations brought about by sexting. Thus, there is a need to stay up-to-date with the apps that adolescents use; discuss sexuality in a professional way (e.g., Van Ouytsel et al., 2014a, 2014b); have the resources to deal with this reality (e.g., Brown et al., 2009); know what sexting is and what drives adolescents to participate in it (e.g., Frankel et al., 2018); and be aware of the ensuing legal and moral obligations (e.g., Schubert & Wurf, 2014). From this perspective, sexting regulations and protocols should be developed in order to provide professionals with a common ground for dealing with this phenomenon. They must be given the necessary tools to act consistently and appropriately, making it easier for pupils to understand the differences between correct and incorrect sexting behavior (e.g., Krieger, 2017; Theodore, 2011). From this perspective and taking into account the other strategies, the adoption of disciplinary or legal measures is crucial in cases where this type of consent is shared non-consensually and where aggressive attitudes are exhibited (e.g., Davis & Schmidt, 2016; Russo & Arndt, 2010); for example, in cases where sexting is associated with bullying (e.g., Van Ouytsel et al., 2014b).

Involvement by the entire education community and potential participants is a key aspect when it comes to fostering coherent and stable actions across the different settings that young people move within and between, thus joining efforts and strategies to tackle this phenomenon (e.g., Frankel et al., 2018).

The peer group risk factors for sexting also play an important role among adolescents. During adolescence, social status is particularly important (Chalfen, 2009; Ling, 2004), and sexting offers an opportunity to become more popular (Gewirtz-Meydan et al., 2018). It may be seen as a strategic move for adolescents to gain popularity among peers (Baumgartner et al., 2015). Thus, encouraging reflection on social pressure and the need for popularity, as well as being critical toward the content received via the Internet, is fundamental (e.g., Ahern & Mechling, 2013; Wolak et al., 2012). Similarly, the false beliefs that adolescents hold about sexting and the notion that all young people engage in this mainstream phenomenon should also be challenged. Adolescents believe that the messages circulating within their immediate surroundings and the media influence their predisposition to develop sexting attitudes, viewing this phenomenon as a normal practice (Smith et al., 2014). The perceptions and experiences of adolescents also make for a good starting point according to the literature, allowing us to understand the whys and hows of their behavior in order to respond to young people’s actual needs (e.g., Livingstone & Görzig, 2014; Murray, 2014).

Efforts to improve the school environment are also reported to have a positive effect on resolving difficult situations associated with sexting. Some examples of good coexistence practices include: maintaining high expectations for student performance; offering pupils the opportunity to participate and contribute in class, at school, and in the education community (e.g., West et al., 2014); implementing peer education/coaching (e.g., Ferrari et al., 2016; Siegle, 2010); and promoting student safety at school, for example, by adopting measures that encourage them to report worrying cases of sexting and other negative behaviors without fear of retaliation (e.g., Gregg et al., 2018; McEachern et al., 2012). Furthermore, when responding to the needs of young people, it is important to cater for diversity and to adopt specific measures that acknowledge, address, and integrate the particularities of vulnerable groups (e.g., Brown et al., 2009; Livingstone & Görzig, 2014).

Finally, in addition to the discussed lines of action, recommendations are made to address this phenomenon early on in the school cycle, likely because the use of virtual networks increases gradually until the age of 13, when it comes into more general use (Garmendia et al., 2016). Furthermore, sexting is characterized by the developmental stage of adolescents’ first romantic or sexual relationships (Fox & Warber, 2013; Van Ouytsel et al., 2016), which highlights the importance of educating young people and relying upon strategies suitable for minors at an early age. Fear tactics and abstinence should also be avoided, as they can make young people increasingly more interested in this practice, without giving them alternative approaches (Gómez & Ayala, 2014). In addition, they do not accurately represent the sexual reality of our contemporary society, preventing us from suggesting strategies to dissuade the negative consequences of sexting among young people (e.g., Döring, 2014). It is also advisable not to judge the victims and individuals who engage in this practice, but rather those who inflict harm and forward content without consent (e.g., Wood et al., 2015). Lastly, evaluation plays a significant role when addressing sexting in order to understand the reality within the corresponding context (e.g., Barrense-Dias et al., 2017; Davis & Schmidt, 2016) and to promote evidence-based practices. Drawing on measurable objectives and considering the definition used is also important. This would enable us to determine the impact and effectiveness of the strategies, so they could act as support mechanisms for professionals working with minors on a daily basis (e.g., Lim et al., 2016; Livingstone & Smith, 2014).

This systematic review does present some limitations. Studies addressing this topic may not have been considered for the following reasons: Sexting was identified through another term; sexting was implicitly covered in intervention programs about other phenomena; or the full article could not be accessed. It is also possible that effective actions are still under development, currently at the “to be published” stage or on the lookout to be published. Future research could build on this review by including studies from other databases, and more comparative studies and further analyses into the nature and characteristics of sexting from an educational point of view would prove useful. There is also a need to evaluate the strategies and actions used to address sexting, with the aim to design and implement evidence-based initiatives that equip schools and teaching staff with effective tools to prevent and tackle the potential risks associated with this phenomenon.

## Appendix: General information on the articles included

**Table:**

No.	Author(s)	Country	Definition of sexting	Evidence of action	Areas of action	Lines of action	Evaluation
1	Diliberto & Mattey (2009)	USA	Sending, receiving and forwarding sexually suggestive pictures or text messages on mobile phones	Proposes specific strategies	1. School 3. Family through school	1. Promoting safe and healthy use of ICT, the Internet and social networks 1, 3. Developing specific sexting programs 1, 3. Raising awareness about the consequences and risks of sexting	No
2	Manzo (2009)	USA	Sharing/exchanging sexually explicit/suggestive pictures on digital devices	Proposes specific strategies	1. School 3. Family through school	1. Training professionals 1, 3. Raising awareness about the consequences and risks of sexting	No
3	Brown et al. (2009)	USA	Sharing/exchanging sexually suggestive or explicit text messages, pictures on social networks or mobile phones	Proposes specific strategies	1. School 3. Family through school	1. Developing measures adapted to vulnerable groups 1. Promoting safe and healthy use of ICT, the Internet and social networks 1. Training professionals 3. Incorporating information about sexting into sex education programs	No
4	Boucek (2009)	USA	Sending sexually suggestive or explicit pictures on mobile phones, computers or another digital device	Proposes specific strategies	1. School 7. Legal advice 9. Technology experts	1, 7, 9. Developing rules and implementing protocols	No
5	Taylor (2009)	USA	Sending sexually suggestive or explicit pictures, generally on mobile phones	Proposes specific strategies	1. School 3. Family through school 8. Law enforcement	1. Training professionals 1, 3, 8. Raising awareness about the consequences and risks of sexting	No
6	Siegle (2010)	USA	Sending sexually suggestive or explicit pictures on the Internet or mobile phones	Proposes specific strategies	1. School 3. Family through school 7. Legal advice	1. Promoting safe and healthy use of ICT, the Internet and social networks 1. Improving the school environment 1, 7. Developing rules and implementing protocols 1. Applying disciplinary or legal measures Training professionals 1, 3. Developing specific sexting programs 1. Encouraging coherence between the different parties involved	No
7	Russo & Arndt (2010)	USA	Sending sexually explicit pictures or text messages on mobile phones or other mobile devices	Proposes specific strategies	1. School 3. Family through school	1. Developing rules and implementing protocols 1. Training professionals 1, 3. Developing specific sexting programs 1, 3. Raising awareness about the consequences and risks of sexting 1. Encouraging coherence between the different parties involved 1. Applying disciplinary or legal measures 1. Promoting safe and healthy use of ICT, the Internet and social networks	No
8	Skarbek & Mooney (2011)	USA	Sending sexually explicit pictures or messages on mobile phones	Recommends acting, but does not provide any specific strategy	1. School 2. Family		No
9	Walker et al. (2011)	Australia	Sending and receiving sexually explicit pictures on mobile phones	Proposes specific strategies	1. School	1. Promoting sexual ethics 1. Considering the ideas and experiences of adolescents	No
10	Segool & Crespi (2011)	USA	--	Recommends acting, but does not provide any specific strategy	1. School		No
11	Dessoff (2011)	USA	--	Recommends acting, but does not provide any specific strategy	1. School		No
12	Theodore (2011)	USA	Sending sexually suggestive or explicit pictures or messages on mobile phones or the Internet	Proposes specific strategies	1. School	1. Raising awareness about the consequences and risks of sexting 1. Incorporating information about sexting into sex education programs 1. Considering the ideas and experiences of adolescents 1. Developing specific sexting programs 1. Developing rules and implementing protocols 1. Applying disciplinary or legal measures	No
13	Rice et al. (2012)	USA	Sending and receiving sexually explicit pictures or messages on mobile phones	Proposes specific strategies	1. School	1. Incorporating information about sexting into sex education programs 1. Promoting safe and healthy use of ICT, the Internet and social networks 1. Promoting sexual ethics 1. Developing measures adapted to vulnerable groups	No
14	Temple et al. (2012)	USA	Sending sexually explicit pictures	Proposes specific strategies	4. Health	4. Incorporating information about sexting into sex education programs 4. Raising awareness about the consequences and risks of sexting	No
15	Kopecký (2012)	Czech Republic	Sharing/exchanging sexually explicit text messages, pictures or videos by electronic means	Proposes specific strategies	1. School	1. Developing specific sexting programs	No
16	Albury & Crawford (2012)	Australia	Sending sexually suggestive or explicit pictures on mobile phones	Proposes specific strategies	6. Policies	6. Promoting sexual ethics 6. Considering the ideas and experiences of adolescents	No
17	Strasburger et al. (2012)	USA	Sharing/exchanging sexually explicit pictures on mobile phones	Proposes specific strategies	1. School	1. Incorporating information about sexting into sex education programs 1. Promoting safe and healthy use of ICT, the Internet and social networks 1. Developing rules and implementing protocols	No
18	McEachern et al. (2012)	USA	Sending sexually explicit pictures or messages using digital and electronic devices	Proposes specific strategies	1. School 2. Family 3. Family through school 6. Policies 8. Law enforcement	1. Applying disciplinary or legal measures 1, 3. Developing specific sexting programs 1. Improving the school environment 1. Developing rules and implementing protocols 1. Raising awareness about the consequences and risks of sexting 1. Training professionals 1, 2, 6, 8. Encouraging coherence between the different parties involved 1. Promoting safe and healthy use of ICT, the Internet and social networks	No
19	Dake et al. (2012)	USA	--	Proposes specific strategies	1. School 2. Family 4. Health	1, 2, 4. Developing specific sexting programs 1. Incorporating information about sexting into sex education programs	No
20	Uhler & Smith (2012)	USA	--	Proposes specific strategies	1. School	1. Applying disciplinary or legal measures	No
21	Sadhu (2012)	USA	--	Proposes specific strategies	4. Health 5. Family through health	4. Training professionals 4, 5. Developing specific sexting programs 4, 5. Raising awareness about the consequences and risks of sexting	No
22	Wolak et al. (2012)	USA	Sending sexually explicit pictures	Recommends acting, but does not provide any specific strategy	1. School 2. Family 4. Health 6. Policies		No
23	Mitchell et al. (2012)	USA	Sending, receiving and forwarding sexually suggestive or explicit pictures, videos or text messages on mobile phones, the Internet or by other electronic means	Proposes specific strategies	1. School	1. Raising awareness about the consequences and risks of sexting 1. Developing specific sexting programs	No
24	Hinduja & Patchin (2012)	USA	--	Proposes specific strategies	1. School	1. Improving the school environment 1. Developing rules and implementing protocols	No
25	Ahern & Mechling (2013)	USA	Sending, receiving and forwarding sexually suggestive or explicit pictures or text messages on mobile phones	Proposes specific strategies	2. Family 4. Health 10. All areas	2, 10. Encouraging coherence between the different parties involved 4. Raising awareness about the consequences and risks of sexting 4. Promoting safe and healthy use of ICT, the Internet and social networks 4. Working on the risk factors associated with the peer group	No
26	Fenaughty & Harré (2013)	New Zealand	Sharing/exchanging sexual material via electronic means	Proposes specific strategies	1. School	1. Promoting safe and healthy use of ICT, the Internet and social networks 1. Raising awareness about the consequences and risks of sexting	No
27	Martín et al. (2013)	Spain	--	Presents intervention	1. School	1. Developing specific sexting programs 1. Promoting safe and healthy use of ICT, the Internet and social networks	Yes
28	Strassberg et al. (2013)	USA	Sending and receiving sexually explicit pictures on mobile phones	Proposes specific strategies	1. School 2. Family 6. Policies 8. Law enforcement	1, 2, 6, 8. Training professionals 1. Developing specific sexting programs 1. Promoting safe and healthy use of ICT, the Internet and social networks	No
29	Van Ouytsel et al. (2014a)	Belgium	Sending sexually suggestive or explicit pictures on the Internet or mobile phones	Proposes specific strategies	1. School	1. Applying disciplinary or legal measures	No
30	Wurtele & Miller-Perrin (2014)	USA	Sending sexually suggestive and explicit pictures, videos and text messages via mobile phones or web cams	Proposes specific strategies	1. School 2. Family 3. Family through school 4. Health 6. Policies 9. Technology experts	1. Promoting safe and healthy use of ICT, the Internet and social networks 1. Developing specific sexting programs 1. Working on the risk factors associated with the peer group 1. Raising awareness about the consequences and risks of sexting 1. Considering the ideas and experiences of adolescents 1. Developing measures adapted to vulnerable groups 1. Promoting sexual ethics 1. Incorporating sexting into preventive programs that tackle other associated risks 1. Incorporating information about sexting into sex education programs 1, 2, 3, 4, 6, 9. Encouraging coherence between the different parties involved	Recommends
31	Kenny (2014)	USA	--	Proposes specific strategies	1. School 2. Family	1, 2. Encouraging coherence between the different parties involved 1. Incorporating information about sexting into sex education programs 1. Developing specific sexting programs	Recommends
32	Livingstone & Görzig (2014)	United Kingdom	Sending and receiving sexually suggestive or explicit pictures or text messages on the Internet	Proposes specific strategies	1. School 3. Family through school	1. Developing measures adapted to vulnerable groups 1. Raising awareness about gender roles and stereotypes 1, 3. Promoting sexual ethics 1. Developing specific sexting programs 1. Incorporating information about sexting into sex education programs 1. Considering the ideas and experiences of adolescents	No
33	Houck et al. (2014)	USA	Sending sexually explicit pictures or messages	Proposes specific strategies	1. School 2. Family 4. Health 7. Family through health	1, 2, 4, 7. Promoting safe and healthy use of ICT, the Internet and social networks 4. Incorporating information about sexting into sex education programs	No
34	Döring ( 2014)	Germany	Sharing/exchanging sexual pictures on mobile phones or the Internet	Proposes specific strategies	1. School 4. Health 6. Policies	1. Raising awareness about the consequences and risks of sexting 1. Developing measures adapted to vulnerable groups 1. Promoting sexual ethics 1. Working on the risk factors associated with the peer group 1. Improving the school environment 1. Considering the ideas and experiences of adolescents 1. Raising awareness about gender roles and stereotypes 1, 4, 6. Training professionals	Recommends
35	Powell & Henry (2014)	Australia	Sharing/exchanging sexually explicit text messages, pictures or videos on mobile phones or social media	Proposes specific strategies	1. School 6. Policies	1, 6. Considering the ideas and experiences of adolescents 1, 6. Promoting sexual ethics 1, 6. Working on the risk factors associated with the peer group 6. Applying disciplinary or legal measures	No
36	Rice et al. (2014)	USA	Sending and receiving sexually explicit pictures or messages on mobile phones	Proposes specific strategies	1. School 2. Family 4. Health	1, 2, 4. Incorporating information about sexting into sex education programs 4. Training professionals 4. Developing specific sexting programs 2. Promoting safe and healthy use of ICT, the Internet and social networks	No
37	Karaian (2014)	Canada	Sharing/exchanging sexual text messages or pictures on mobile phones or by other electronic means	Proposes specific strategies	10. All areas/general	10. Raising awareness about gender roles and stereotypes	No
38	Smith et al. (2014)	United Kingdom	Sending, receiving or forwarding sexually explicit pictures or messages by electronic means, mainly between mobile phones	Proposes specific strategies	1. School 2. Family 6. Policies	1. Promoting safe and healthy use of ICT, the Internet and social networks 1, 2, 6. Developing rules and implementing protocols 1. Developing measures adapted to vulnerable groups 1. Raising awareness about gender roles and stereotypes	Recommends
39	Hillman et al. (2014)	Australia	Sending sexually explicit pictures, videos or messages	Proposes specific strategies	6. Policies		No
40	Mura et al. (2014)	Italy	--	Proposes specific strategies	1. School	1. Promoting safe and healthy use of ICT, the Internet and social networks 1. Training professionals	No
41	Srinivas et al. (2014)	USA	--	Proposes specific strategies	1. School 3. Family through school 4. Health 6. Policies	1, 4, 6. Training professionals 1, 3. Developing specific sexting programs	No
42	Livingstone & Smith (2014)	United Kingdom	--	Proposes specific strategies	10. All areas/general		Recommends
43	West et al. (2014)	Peru	Sending and receiving sexually suggestive or explicit text messages	Proposes specific strategies	1. School 3. Family through school 4. Health 5. Family through health	1, 3, 4, 5. Promoting safe and healthy use of ICT, the Internet and social networks 1, 3, 4, 5. Developing specific sexting programs 1. Improving the school environment	No
44	Soriano-Ayala & González-Jiménez (2014)	Spain	Sending sexually suggestive or explicit pictures, videos or text messages on mobile phones	Proposes specific strategies	1. School	1. Promoting safe and healthy use of ICT, the Internet and social networks 1. Considering the ideas and experiences of adolescents	No
45	Schubert & Wurf (2014)	Australia	Sending sexually explicit pictures, videos or messages. Although frequently associated with mobile phones, sexting does not just take the form of a text or multimedia message, but can also be via email, publication on user-generated sites such as YouTube or Flickr, uploaded on social networks and live streaming on a web cam	Proposes specific strategies	1. School	1. Training professionals 1. Promoting sexual ethics 1. Incorporating information about sexting into sex education programs 1. Raising awareness about gender roles and stereotypes	No
46	Van Ouytsel et al. (2014b)	Belgium	Sending sexually suggestive or explicit pictures, videos or text messages by text, smart phone, camera phones and Web 2.0	Proposes specific strategies	1. School 3. Family through school 7. Legal advice	1, 7. Developing rules and implementing protocols 1. Encouraging coherence between the different parties involved 1. Incorporating sexting into preventive programs that tackle other associated risks 1. Improving the school environment 1. Training professionals 1, 3. Developing specific sexting programs 1. Working on the risk factors associated with the peer group 1, 3. Promoting safe and healthy use of ICT, the Internet and social networks 1. Incorporating information about sexting into sex education programs 1. Promoting sexual ethics 1. Raising awareness about gender roles and stereotypes 1. Considering the ideas and experiences of adolescents 1. Applying disciplinary or legal measures	Recommends
47	Ybarra & Mitchell (2014)	USA	Sending sexually explicit messages via any means: in person, on the Internet, by mobile phone, text message or any other way	Proposes specific strategies	1. School 2. Family 4. Health 8. Law enforcement	1. Incorporating sexting into preventive programs that tackle other associated risks 1. Incorporating information about sexting into sex education programs 1, 2, 4, 8. Training professionals	No
48	Murray (2014)	USA	Sharing/exchanging sexually explicit text messages, pictures or videos by mobile phone or other electronic devices	Proposes specific strategies	1. School	1. Raising awareness about the consequences and risks of sexting 1. Considering the ideas and experiences of adolescents 1. Developing rules and implementing protocols 1. Developing specific sexting programs	No
49	Wood et al. (2015)	United Kingdom	Sending and receiving sexually explicit pictures or messages	Proposes specific strategies	1. School	1. Working on the risk factors associated with the peer group 1. Raising awareness about gender roles and stereotypes 1. Considering the ideas and experiences of adolescents 1. Promoting sexual ethics	No
50	Pellai et al. (2015)	Italy	Sharing/exchanging sexually explicit pictures or videos	Proposes specific strategies	1. School 3. Family through school	1. Incorporating information about sexting into sex education programs 1. Training professionals 1, 3. Developing specific sexting programs 1. Incorporating sexting into preventive programs that tackle other associated risks	No
51	Eugene (2015)	USA	Sending sexually suggestive pictures	Proposes specific strategies	1. School	1. Developing specific sexting programs 1. Considering the ideas and experiences of adolescents 1. Incorporating information about sexting into sex education programs	No
52	Van Ouytsel et al. (2015)	Belgium	Sharing/exchanging sexually explicit content via mobile phones and the Web 2.0, such as social media	Proposes specific strategies	1. School 3. Family through school 4. Health 7. Legal advice	1. Working on the risk factors associated with the peer group 1. Incorporating sexting into preventive programs that tackle other associated risks 1. Incorporating information about sexting into sex education programs 1. Promoting safe and healthy use of ICT, the Internet and social networks 1. Training professionals 1, 4. Raising awareness about the consequences and risks of sexting 1, 7. Developing rules and implementing protocols 1, 3. Developing specific sexting programs 1. Encouraging coherence between the different parties involved	Recommends
53	Kopecký & Szotkowski (2015)	Czech Republic	Sharing/exchanging intimate material via the Internet	Proposes specific strategies	1. School 3. Family through school	1. Promoting safe and healthy use of ICT, the Internet and social networks 1. Developing specific sexting programs 1, 3. Encouraging coherence between the different parties involved	No
54	Kopecký et al. (2015)	Czech Republic	Sharing/exchanging intimate material via the Internet	Presents intervention	1. School	1. Promoting safe and healthy use of ICT, the Internet and social networks 1. Improving the school environment 1. Developing specific sexting programs 1. Incorporating sexting into preventive programs that tackle other associated risks	No
55	Kopecký (2015)	Slovak Republic	Sending and receiving sexually suggestive or explicit pictures, videos and text messages	Proposes specific strategies	1. School	1. Raising awareness about the consequences and risks of sexting 1. Developing specific sexting programs 1. Training professionals	No
56	Patrick et al. (2015)	Australia	Sending and receiving sexually explicit pictures, videos and messages This also includes sending and receiving sexually suggestive pictures or videos	Proposes specific strategies	1. School	1. Promoting sexual ethics 1. Promoting safe and healthy use of ICT, the Internet and social networks 1. Raising awareness about the consequences and risks of sexting	No
57	Choi et al. (2016)	USA	Sending and receiving sexually explicit pictures via text message, email or Snapchat	Proposes specific strategies	1. School	1. Promoting sexual ethics	No
58	O’Keeffe (2016)	USA	--	Proposes specific strategies	4. Health 5. Family through health	4, 5. Promoting safe and healthy use of ICT, the Internet and social networks	No
59	Ahern et al. (2016)	USA	--	Proposes specific strategies	1. School 3. Family through school 4. Health	1, 3. Developing specific sexting programs 1, 3. Raising awareness about the consequences and risks of sexting 1. Encouraging coherence between the different parties involved 1, 4. Training professionals	Recommends
60	American College of Obstetricians & Gynecologists (2016)	USA	Sending sexually suggestive or explicit pictures or messages on mobile phones	Proposes specific strategies	1. School 2. Family 4. Health 5. Family through health	4. Raising awareness about the consequences and risks of sexting 4, 5. Promoting safe and healthy use of ICT, the Internet and social networks 1, 2, 4. Encouraging coherence between the different parties involved	No
61	Dobson & Ringrose (2016)	United Kingdom & Australia	Sharing/exchanging sexually explicit or suggestive text messages or pictures on mobile phones and social networks	Proposes specific strategies	1. School 6. Policies	1. Raising awareness about gender roles and stereotypes 1. Promoting safe and healthy use of ICT, the Internet and social networks 1. Promoting sexual ethics 1, 6. Training professionals	No
62	Lim et al. (2016)	Australia	Sending and receiving sexually explicit pictures on the Internet or mobile phones	Proposes specific strategies	1. School	1. Promoting sexual ethics 1. Working on the risk factors associated with the peer group 1. Raising awareness about the consequences and risks of sexting 1. Incorporating information about sexting into sex education programs	Recommends
63	Ferrari et al. (2016)	Italy	--	Presents intervention	1. School 10. All areas/general	1, 10. Improving the school environment 1, 10. Promoting safe and healthy use of ICT, the Internet and social networks	No
64	Loveless (2016)	USA	Sending sexually suggestive or explicit pictures or messages on mobile phones	Proposes specific strategies	1. School 2. Family 4. Health 5. Family through health	4. Raising awareness about the consequences and risks of sexting 4, 5. Promoting safe and healthy use of ICT, the Internet and social networks 1, 2, 4. Encouraging coherence between the different parties involved	No
65	Davis & Schmidt (2016)	USA	Sending, receiving and forwarding sexually explicit pictures and messages on mobile phones, the computer or another digital device	Presents intervention	1. School 3. Family through school 7. Legal advice	1. Encouraging coherence between the different parties involved 1, 7. Developing rules and implementing protocols 1. Developing measures adapted to vulnerable groups 1. Promoting safe and healthy use of ICT, the Internet and social networks 1. Training professionals 1, 3. Developing specific sexting programs 1. Applying disciplinary or legal measures	Recommends
66	Palop et al. (2016)	Spain	Sending, receiving or forwarding sexually explicit or suggestive pictures, videos or messages via mobile phones, the Internet or social networks	Presents intervention	1. School	1. Raising awareness about the consequences and risks of sexting 1. Promoting safe and healthy use of ICT, the Internet and social networks 1. Developing specific sexting programs	Yes
67	Lee et al. (2016)	South Korea	Sending sexually explicit pictures, videos or messages via the mobile phone	Proposes specific strategies	1. School	1. Raising awareness about the consequences and risks of sexting 1. Working on the risk factors associated with the peer group	No
68	Ease (2016)	--	--	Proposes specific strategies	1. School	1. Promoting safe and healthy use of ICT, the Internet and social networks 1. Raising awareness about the consequences and risks of sexting 1. Developing rules and implementing protocols	No
69	Speno (2016)	USA	Sending, receiving and forwarding sexually suggestive or explicit pictures or text messages on mobile phones or other mobile devices	Proposes specific strategies	1. School	1. Raising awareness about gender roles and stereotypes 1. Developing specific sexting programs	No
70	Barrense-Dias et al. (2017)	Switzerland	--	Proposes specific strategies	10. All areas/general	10. Incorporating sexting into preventive programs that tackle other associated risks 10. Working on the risk factors associated with the peer group 10. Developing specific sexting programs	No
71	Anastassiou (2017)	United Kingdom	Sending and receiving sexually explicit pictures on the Internet	Recommends acting, but does not provide any specific strategy	6. Policies 10. All areas/general		No
72	Rodríguez-Castro et al. (2017)	Spain	Sending, receiving and forwarding sexually suggestive or explicit pictures, videos or text messages	Proposes specific strategies	1. School	1. Raising awareness about gender roles and stereotypes	No
73	Villacampa (2017)	Spain	Sending, receiving and forwarding sexually suggestive or explicit pictures, videos or text messages	Proposes specific strategies	1. School 3. Family through school	1. Raising awareness about gender roles and stereotypes 1. Raising awareness about the consequences and risks of sexting 1. Training professionals 1, 3. Developing specific sexting programs	No
74	Wachs et al. (2017)	Germany, Netherlands & Thailand	Sending sexually suggestive or explicit pictures via the Internet	Proposes specific strategies	1. School	1. Raising awareness about the consequences and risks of sexting 1. Promoting safe and healthy use of ICT, the Internet and social networks 1. Developing specific sexting programs 1. Promoting sexual ethics	No
75	Gámez-Guadix et al. (2017)	Spain	Sending sexually suggestive or explicit pictures, videos or text messages	Proposes specific strategies	1. School	1. Promoting safe and healthy use of ICT, the Internet and social networks 1. Raising awareness about the consequences and risks of sexting 1. Developing measures adapted to vulnerable groups	No
76	Krieger (2017)	Canada	Sending or receiving self-produced sexual material on mobile devices and social media sites	Proposes specific strategies	1. School	1. Raising awareness about gender roles and stereotypes 1. Developing rules and implementing protocols	No
77	Strassberg et al. (2017)	USA	Sending, receiving or forwarding sexually explicit material on mobile phones	Presents intervention	1. School	1. Raising awareness about the consequences and risks of sexting	Yes
78	Albury et al. (2017)	USA	--	Proposes specific strategies	1. School	1. Considering the ideas and experiences of adolescents 1. Promoting sexual ethics 1. Developing specific sexting programs 1. Incorporating information about sexting into sex education programs 1. Promoting safe and healthy use of ICT, the Internet and social networks 1. Raising awareness about gender roles and stereotypes	No
79	Delmonico et al. (2017)	USA	--	Recommends acting, but does not provide any specific strategy	1. School		No
80	Norman (2017)	Canada	Sending, receiving or forwarding sexually explicit or suggestive pictures, videos or messages	Recommends acting, but does not provide any specific strategy	10. All areas/general		No
81	Bhat (2018)	USA & Australia	Sending and receiving sexually suggestive or explicit pictures, videos or messages on mobile phones or social network platforms	Proposes specific strategies	1. School 2. Family	1. Improving the school environment 1. Training professionals 1, 2. Encouraging coherence between the different parties involved 1. Developing rules and implementing protocols 1. Promoting safe and healthy use of ICT, the Internet and social networks 1. Promoting sexual ethics 1. Developing specific sexting programs	No
82	Charteris et al. (2018)	Australia	Sharing/exchanging sexually suggestive or explicit pictures or videos via mobile phones	Proposes specific strategies	1. School 2. Family	1, 2. Promoting safe and healthy use of ICT, the Internet and social networks	No
83	Frankel et al. (2018)	USA	Sharing/exchanging sexually suggestive or explicit pictures	Proposes specific strategies	1. School 3. Family through school	1, 3. Developing specific sexting programs 1. Incorporating information about sexting into sex education programs 1. Training professionals 1. Encouraging coherence between the different parties involved 1. Developing rules and implementing protocols	No
84	Symons et al. (2018)	Belgium	Sending sexually explicit pictures on the Internet or mobile phones	Proposes specific strategies	1. School 10. All areas/general	10. Developing specific sexting programs 1. Incorporating information about sexting into sex education programs 1. Raising awareness about gender roles and stereotypes	No
85	Marume et al. (2018)	Zimbabwe	Sending sexually explicit pictures	Proposes specific strategies	10. All areas/general	10. Incorporating information about sexting into sex education programs 10. Developing specific sexting programs	
86	De Souza & Alves (2018)	Brazil	Sharing/exchanging sexually explicit texts or messages by electronic means	Proposes specific strategies	1. School	1. Incorporating information about sexting into sex education programs 1. Working on the risk factors associated with the peer group 1. Promoting sexual ethics 1. Developing specific sexting programs	No
87	Wolak et al. (2018)	USA	--	Proposes specific strategies	1. School 3. Family through school	1. Developing specific sexting programs 1. Promoting sexual ethics 1, 3. Promoting safe and healthy use of ICT, the Internet and social networks 1. Raising awareness about gender roles and stereotypes	No
88	Stanley et al. (2018)	United Kingdom	Sending and receiving sexually explicit pictures or messages on mobile phones or social media platforms	Proposes specific strategies	1. School	1. Working on the risk factors associated with the peer group 1. Raising awareness about gender roles and stereotypes 1. Incorporating information about sexting into sex education programs	No
89	Madigan et al. (2018)	Canada, Belgium & USA	Sending, receiving or forwarding sexually explicit pictures, videos or text messages	Proposes specific strategies	1. School 2. Family 4. Health	1. Promoting safe and healthy use of ICT, the Internet and social networks 1. Promoting sexual ethics	No
90	Gregg et al. (2018)	USA	Sending and receiving suggestive or explicit pictures, messages or videos	Proposes specific strategies	1. School 2. Family	1. Improving the school environment 1. Raising awareness about the consequences and risks of sexting 2. Promoting safe and healthy use of ICT, the Internet and social networks 1. Incorporating information about sexting into sex education programs 1. Working on the risk factors associated with the peer group 1. Developing specific sexting programs	No
91	Laguado et al. (2018)	Colombia	Sending pictures or videos containing content that is sexual to a certain extent, filmed or recorded by the protagonists thereof, on a mobile phone	Proposes specific strategies	1. School	1. Incorporating information about sexting into sex education programs 1. Developing specific sexting programs	No

^1^The numbers in “lines of action” refer to the numbers in “areas of action” to which they correspond.
